# Magnetization Manipulation of a Flexible Magnetic Sensor by Controlled Stress Application

**DOI:** 10.1038/s41598-018-34036-z

**Published:** 2018-10-25

**Authors:** Joon-Hyun Kwon, Won-Young Kwak, Beong Ki Cho

**Affiliations:** School of Materials Science and Engineering, Gwangju Institute of Science and Technology, Gwangju, 61005 Republic of Korea

## Abstract

Spin-based electronic devices on polymer substrates have been intensively investigated because of several advantages in terms of weight, thickness, and flexibility, compared to rigid substrates. So far, most studies have focused on maintaining the functionality of devices with minimum degradation against mechanical deformation, as induced by stretching and bending of flexible devices. Here, we applied repetitive bending stress on a flexible magnetic layer and a spin-valve structure composed of Ta/NiFe/CoFe/Cu/Ni/IrMn/Ta on a polyimide (PI) substrate. It is found that the anisotropy can be enhanced or weakened depending upon the magnetostrictive properties under stress. In the flat state after bending, due to residual compressive stress, the magnetic anisotropy of the positive magnetostrictive free layer is weakened while that of the pinned layer with negative magnetostriction is enhanced. Thus, the magnetic configuration of the spin-valve is appropriate for use as a sensor. Through the bending process, we design a prototype magnetic sensor cell array and successfully show a sensing capability by detecting magnetic microbeads. This attempt demonstrates that appropriate control of stress, induced by repetitive bending of flexible magnetic layers, can be effectively used to modify the magnetic configurations for the magnetic sensor.

## Introduction

Electronic devices have become ultra-thin, ultra-lightweight, and flexible for application as wearable and portable devices, such as paper-like displays^1,2^, skin-like electronic devices^3,4^, flexible solar cells and light emitting diodes^5–7^, and molecular-scale electronic devices^8^. These devices are typically fabricated on various polymer substrates, such as polyimide (PI), polydimethylsiloxane (PDMS), polyester and polyethylene terephthalate (PET). In particular, PI film is widely used because of desirable thermosetting properties with high thermal and chemical stabilities. As a result, in contrast to devices based on rigid silicon-based substrates, the influence of the flexibility of polymer substrates on the performance of electronic devices has been investigated. One of the main issues for flexible electronic devices is focused on the reproducibility and retention of device characteristics because of mechanical deformation due to stretching and bending. For magnetic devices, the effects of strain on flexible magnetic memory^9–11^ and sensors^12–14^ as well as on a single magnetic layer have also been studied^15,16^. Therefore, zig-zag or wrinkled types and pre-strained structures have been suggested to reduce the influence of external stress and to make devices robust against large strain^14,17–20^. On the other hand, since magnetic materials have inverse magnetostriction effect, i.e., a magnetization change induced by external stress, additional attention should be paid to mechanical effects. Therefore, it would be interesting to investigate the effects of stress on the magnetic characteristics of a flexible magnetic film. In this study, we applied an intentional and controlled stress to flexible magnetic devices to induce a desired magnetic configuration for a specific magnetic functionality.

Conventional magnetic spin-valve sensors on a silicon wafer, which are widely used in bio chip applications^21–27^ should show an orthogonal magnetization structure between the free and pinned layers. The orthogonality results in a linear resistance dependence on the applied field, which enables a spin-valve to function as a magnetic sensor. Two methods can be used to realize such an orthogonal magnetization configuration between the free and pinned layers in a spin-valve structure. One is the application of an orthogonal magnetic field using a permanent magnet during deposition of the free and pinned ferromagnetic layers before the film is patterned into a specific shape with high aspect ratio for high shape anisotropy. Another method is the use of a post-annealing process for rotation of the pinned layer magnetization^28,29^.

Here, we report an improvement in the magnetoresistance (MR) ratio of a spin-valve structure of Ta/NiFe/CoFe/Cu/Ni/IrMn/Ta on a PI substrate, where NiFe and Ni layers are the free and pinned layers, respectively, by stress application, induced by controlled bending of the flexible layers, without post annealing processing. The MR ratio for a conventional spin-valve of Ni and permalloy on a silicon substrate shows relatively low values of ≈2~3% even if an adjacent antiferromagnetic layer is used for exchange bias with the pinned ferromagnetic layer^30–32^. However, we realized a significantly high MR ratio of 7% by applying a bending stress, which led to enhanced magnetic anisotropy due to an inverse magnetostriction effect. In addition, bending stress induces a linear dependence for resistance on the magnetic field because the magnetostriction coefficients for Ni and permalloy are negative and positive, respectively. With the combination of a high MR ratio and linear behaviour, a spin-valve magnetic sensor with high sensitivity and adjustable sensing range is demonstrated. Finally, a prototype magnetic sensor is fabricated and tested to verify the capability for detection of a generated stray field from magnetic microbeads, as used in bio sensors.

## Experimental Details

### Nanostructure deposition

**Figure 1:**
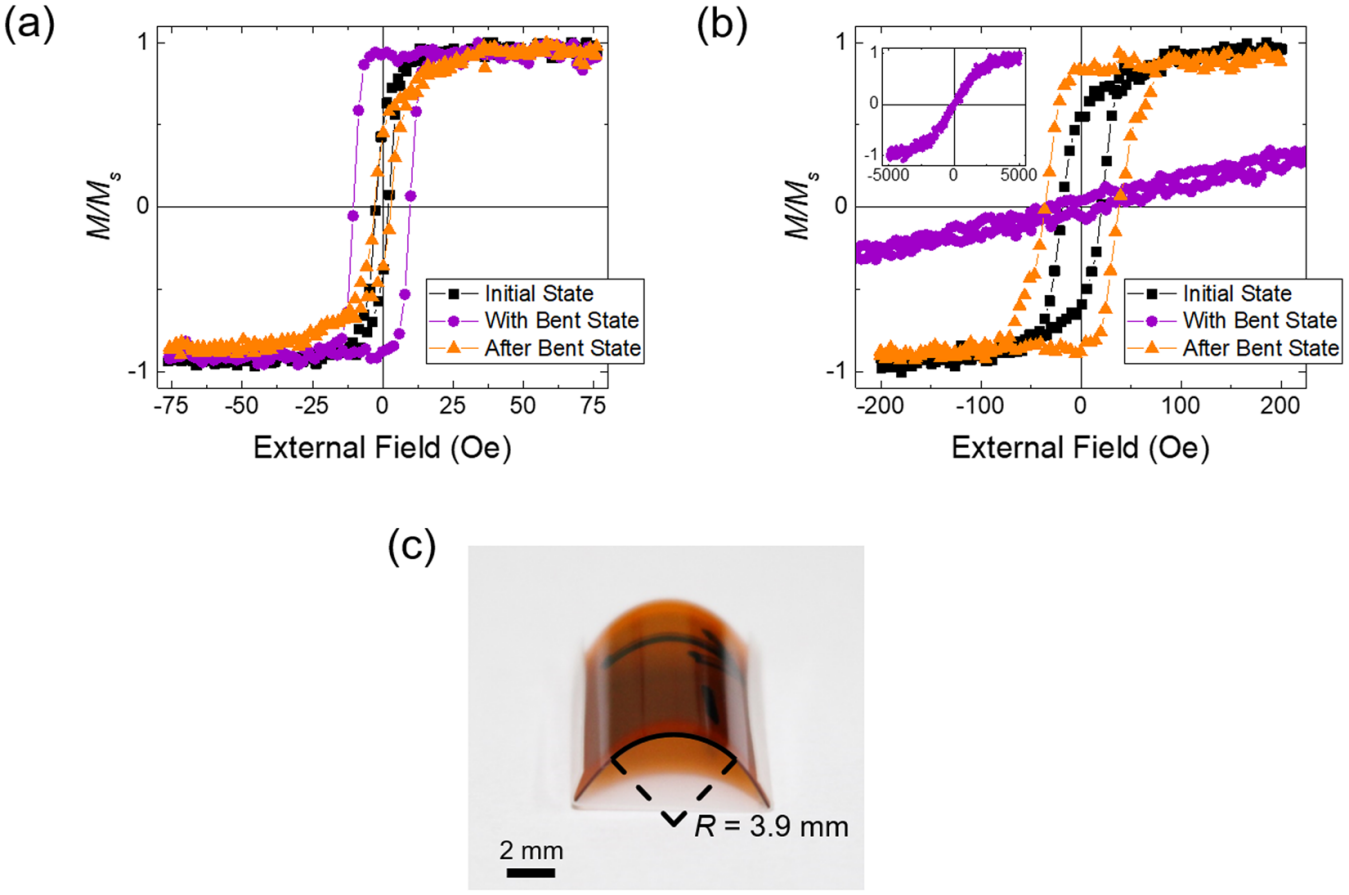
(**a**) and (**b**) Hysteresis loops for NiFe and Ni layers with 4-nm thickness in the initial-flat-state (black square symbols), bent-state (purple circle symbols), and in the flat state after bending (after-flat-state), (orange triangle symbols). Inset of (**b**) shows the full magnetization curve for the Ni layer in the bent-state. (**c**) The magnetic layer on the PI substrate in the downward bent state with the smallest bending radius of 3.9 mm. The black arrow indicates the magnetic easy axis on the back side of the PI substrate.

Spin-valve structures are deposited onto 125-μm thickness of PI substrate using the DC magnetron sputtering system with a base pressure of ≈8 × 10^−9^ Torr. A magnetic field of ≈200 Oe to set the magnetic easy axis was applied during deposition. The spin-valve structure is consist of Ta/Ni_79_Fe_21_/Co_70_Fe_30_/Cu/Ni/Ir_20_Mn_80_/Ta, where Ni_79_Fe_21_/Co_70_Fe_30_ and Ni layers are used as the free layer and pinned layers, respectively. The deposition rates of each layer are 0.182 Å/s for Ta, 0.221 Å/s for Ni_79_Fe_21_, 0.175 Å/s for Co_70_Fe_30_, 0.298 Å/s for Cu, 0.215 Å/s for Ni, and 0.074 Å/s for Ir_20_Mn_80_ at an Ar pressure of 3 mTorr. Magnetostriction coefficients for Co_70_Fe_30_ and Ni_79_Fe_21_ are positive^33^, whereas that for Ni is negative^34^. Due to higher wt.% of iron compared to common permalloy (Ni_80_Fe_20_), Ni_79_Fe_21_ shows characteristics of a stable positive magnetostriction and low *H*_*c*_, as shown in Fig. 1(b). The structure used in the ‘Supporting Information’ is of Ta/Cu/Ir_20_Mn_80_/Ta in order to exclude the influence of ferromagnetic layers.

## Results and Discussion

### The influence of stress application on the magnetic single layer

Figure 1(a) and (b) show the change in magnetic properties for NiFe and Ni layers, respectively, deposited on a flexible PI substrate, for tensile stress applied by bending of the magnetic layers, as shown in Fig. 1(c). Each layer was deposited to a thickness of 4 nm and covered with 1-nm thickness of Ta layer. In the downward bent state, tensile stress is applied along the magnetic easy axis. For the PI film with an area of 12 × 12 mm^2^, the bending radius at the center is 3.9 mm. The stress on the magnetic layer can be calculated by the Stoney equation^35^, which is expressed as ${\sigma }_{f}=1/6R\times \frac{{E}_{s}{d}_{s}^{2}}{(1-{\upsilon }_{s}){d}_{f}}$, where R is the bending radius, *E*_*s*_ i_*s*_ the Young’s modulus of the sub_*s*_trate, *d*_*s*_ is the thickness of substrate, *υ*_*s*_ is Poisson’s ratio for the substrate, and *d*_*f*_ is thickness of the thin film. According to the equation, a stress of 632 GPa is estimated using a Young’s modulus of 2.5 GPa, Poisson’s ratio of 0.34 and thickness of 125 μm for the PI substrate^36^.

For NiFe, magnetic hysteresis reveals a coercive field (*H*_*c*_) of ≈2 Oe and squareness ratio, which is defined as the remanence magnetization/saturation magnetization, of ≈0.53 before bending. The values of *H*_*c*_ and squareness increase to ≈10 Oe and ≈0.97 in the bent state, respectively, in which tensile stress is applied along the magnetic easy axis. On the other hand, for Ni, the changes in *H*_*c*_ and squareness in the bent state are quite different. The hysteresis feature disappears so that the magnetization curve is almost linear in the low field region and the effective anisotropy field, *H*_*k*_, increases significantly to ≈2.7 kOe.

The changes in magnetic hysteresis can be understood in terms of an intrinsic magnetostriction effect. The magnetostriction constant (*λ*_*s*_) for NiFe, which is composed of 79 wt.% of Ni and 21 wt.% of Fe, is +6.5 × 10^−6^ ^37^, while *λ*_*s*_ for Ni is −2.4 × 10^−5^ ^34^ For NiFe with positive *λ*_*s*_, tensile stress will enhance the magnetization, i.e., enforce magnetic anisotropy along the stress and the magnetic easy axis. Thus, *H*_*c*_ and squareness for NiFe will both increase in the bent state, as shown in Fig. 1(a). For Ni with negative *λ*_*s*_, the magnetic anisotropy decreases along the tensile stress and increases over a transverse direction, resulting in the loss of hysteresis and an increase in *H*_*k*_, as shown in the inset of Fig. 1(b). The magnetic anisotropy energy is defined as ${\rm{E}}={K}_{u}si{n}^{2}\theta$, where *K*_*u*_ is the uniaxial anisotropy constant and *θ* is the angle between the magnetic easy axis (or stress axis) and magnetization within the film layer. The effective *K*_*u*_ can be expressed as *K*_1_ for crystal anisotropy, $-1/2{\mu }_{0}{{M}_{s}}^{2}$ for shape anisotropy, and $3/2{\lambda }_{s}\sigma$ for stress anisotropy, where *μ*_0_, *M*_*s*_, and *σ* denote permeability, saturation magnetization, and the external stress, respectively. Shape anisotropy is ignored in this study because the substrate has an aspect ratio of 1 in the plane for both NiFe and Ni. The crystal anisotropy constant for NiFe is −2.2 × 10^3^ dyn/cm^2^ with *K*_1_ of −2.2 × 10^3^ erg/cm^3^ and the stress anisotropy constant is estimated to be + 6.2 × 10^7^ dyn/cm^2^ ^37^, whereas the crystal anisotropy constant for Ni is −4.5 × 10^4^ dyn/cm^2^ with *K*_1_ of −4.5 × 10^4^ erg/cm^3^, and the stress anisotropy constant is −2.3 × 10^8^ dyn/cm^2^ ^34^. Thus, it is found that the effective *K*_*u*_ for stress anisotropy is much larger than crystal anisotropy *K*_1_ for both NiFe and Ni. This means that the magnetization properties can be mainly determined by stress effects, rather than intrinsic crystal isotropy, consistent with the interpretation for the hysteresis change given above.

The magnetic states do not return to their initial states after the stress is released for both NiFe and Ni, as shown in Fig. 1. The hysteresis curve for the NiFe layer in the flat state after the stress is released indicates that the magnetic anisotropy along the stress direction is weakened. On the other hand, the hysteresis curve for the Ni layer indicates that the anisotropy is enhanced. These behaviours can also be understood in terms of an inverse magnetostriction effect. Compressive stress is applied on the NiFe and Ni layers on the way to releasing the tensile stress or residual compressive stress is applied after the stress is released. The final magnetic states persist without further changes. This indicates that although both tensile and compressive stress induced by bending and after bending states of the flexible layer, respectively, affects the magnetization, compressive stress is more important to determine the final magnetization state of the flexible layer compared to tensile stress.

### Change of the magnetic configuration in spin-valve on PI substrate by repetitive bending

**Figure 2:**
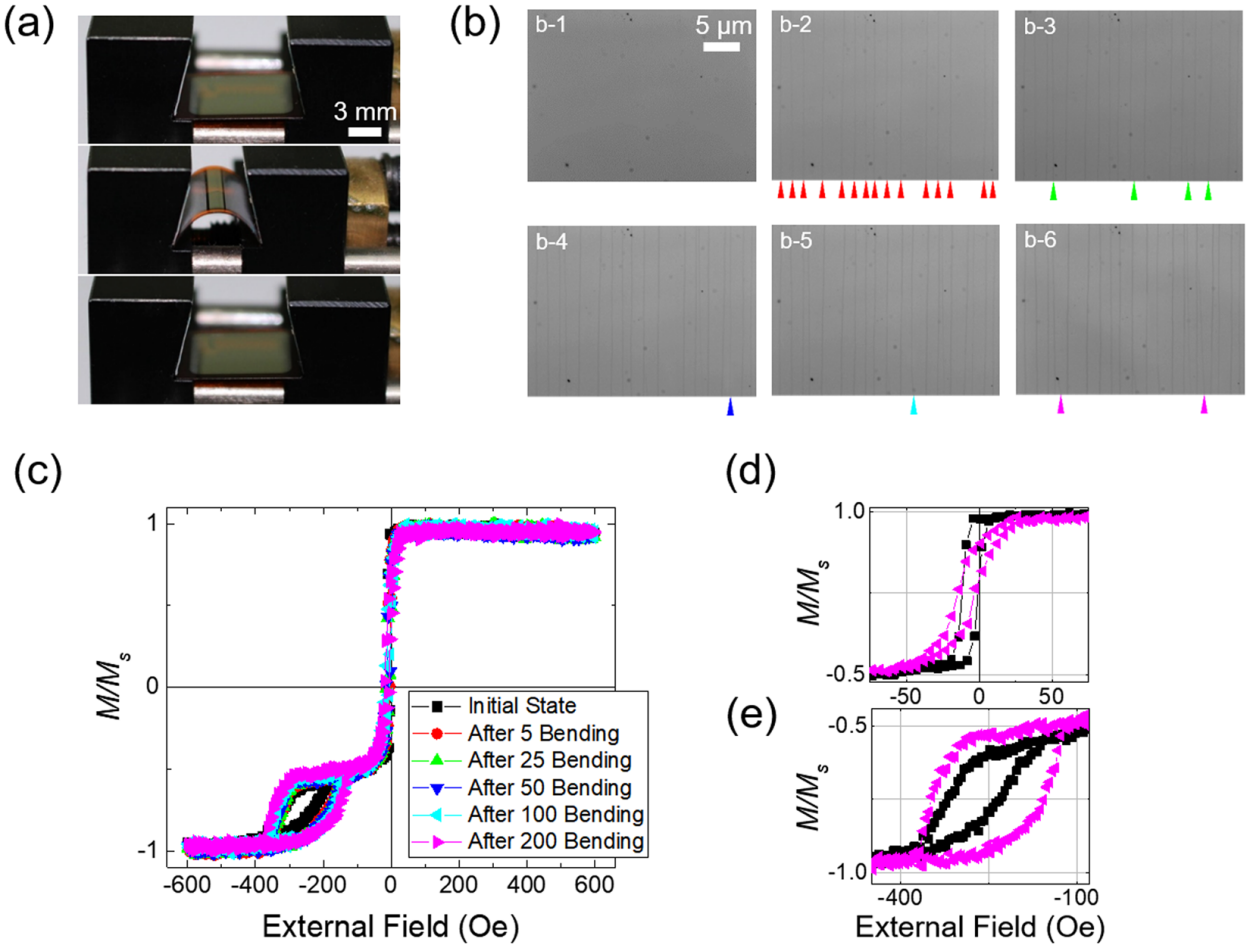
(**a**) The flexible spin-valve film in the bending machine in the initial-flat-state, bent-state, and in the flat state after bending (after-flat-state) from top to bottom. (**b**) Optical images for the top surface of the spin-valve. b-1 is the initial surface, and b-2 to b-6 are the surfaces after 5 (red), 25 (green), 50 (blue), 100 (cyan), and 200-times (pink) bending applied sequentially. Arrows indicate the location of cracks produced after each step. (**c**) Magnetization curves for the flexible spin-valve in the after-flat-state for varying numbers of bending cycles. (**d**) and (**e**) Expanded plots of (**c**) near zero and −250 Oe, respectively, in the initial-flat-state and in the after-flat-state for 200-times bending.

We applied bending-induced stress to the flexible spin-valve structure, which consists of PI substrate/Ta 3/NiFe 4/CoFe 1/Cu 2.8/Ni 4/IrMn 15/Ta 1 (nm). The ferromagnetic NiFe is a free layer, and Ni is a pinned layer by antiferromagnetic IrMn. The thin CoFe sub-layer improves the MR ratio by increasing spin transport at the interface between the free and spacer layer (Cu) while maintaining a small *H*_*c*_ for NiFe^38,39^. The Ta layer at the bottom is used for buffer layers, which improves adhesion to the substrate and the crystallinity of the magnetic layer, with the top layer of Ta used as a capping layer. External stress is repetitively applied to the spin-valve structure along the magnetic easy axis by using a mechanical bending machine, as shown in Fig. 2(a), which enables one to apply the most uniform stress as possible. The three pictures from top to bottom indicate the initial-flat-state, bent-state, and flat state after the bending is released, i.e., after-flat-state, respectively. In the bent-state, the film shows the smallest bending radius of 3.9 mm, which is the same as that in Fig. 1. Figure 2(b) shows optical images for the top surface of the spin-valve before and after 5, 25, 50, 100, and 200-times bending, respectively. All images are taken at the centre of the film surface, where the largest stress is applied due to the bending. A large number of cracks are found on the surface even after 5-times bending at the positions indicated by red arrows. The cracks formed are found to be almost straight and lie along the direction transverse to the bending each separated by a distance of 2~10 μm. The number of cracks gradually increases with the number of bending cycles.

Figure 2(c) shows magnetization curves in terms of the applied field in the after-flat-state with 5, 25, 50, 100, and 200-times repetitive bending. Magnetization reversal for the NiFe free layer occurs near zero field, while the magnetization of the Ni pinned layer is reversed in a range of −400 Oe ≤ *H* ≤ −120 Oe due to exchange coupling with IrMn. Although the thicknesses of the free and pinned layers are identical, the free layer (NiFe) has a larger magnetic moment compared to the pinned layer (Ni) so that the magnetic reversal of free layer near zero field is completed in negative magnetization. The hysteresis loop for the NiFe and Ni layers after 200-times bending is shown together with that obtained for the initial-flat-state for comparison in Fig. 2d,e, respectively. For the NiFe layer, the hysteresis loop changes to be tilted with increased *H*_*k*_ in a low magnetic field range. For the Ni layer, the hysteresis is enhanced, resulting in an increase in *H*_*c*_ and the squareness ratio. These features become monotonically clearer with the number of bending cycles up to 200-times. The changes in the hysteresis loops are consistent with the results shown in Fig. 1, e.g., positive (NiFe) and negative (Ni) magnetostriction effects due to compressive residual stress in the film. This finding indicates that the anisotropy for the pinned and free layers in a spin-valve can be enhanced and weakened, respectively, at the same time by applying a bending stress and stress effect can be accumulated through repetitive bending.

**Figure 3:**
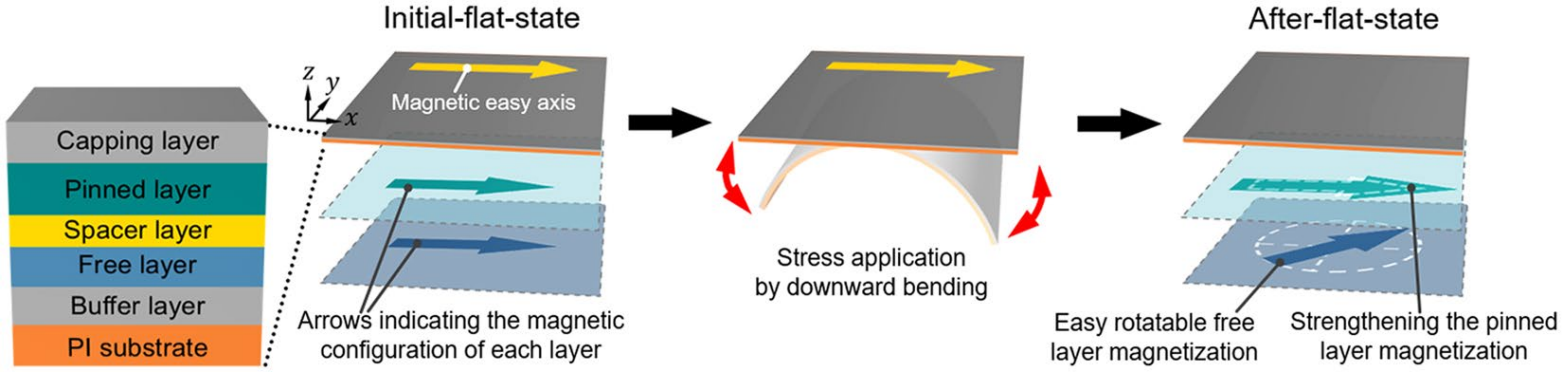
Schematic illustration of magnetic configuration in a spin-valve on flexible PI substrate before and after downward bending stress is applied along the direction of the magnetic easy axis.

Figure 3 shows a schematic illustration for the change of magnetic configuration in a spin-valve by application of downward bending stress, as described in Fig. 2. At initial-flat-state, the magnetic configuration of free (blue plane) and pinned (green plane) layers is parallel each other along the magnetic easy axis, which is established by the applied field during deposition. After downward bending stress is applied along the magnetic easy axis, magnetic anisotropy of the free layer decreases and relatively free to rotate at after-flat-state with the applied field but the anisotropy of the pinned layer is increases. Repetitive bending stress by the mechanical bending machine accumulates these effects and, as a result, the magnetic configuration of the free and pinned layers is settled down after 200-times bending, as shown in Fig. 2.

**Figure 4:**
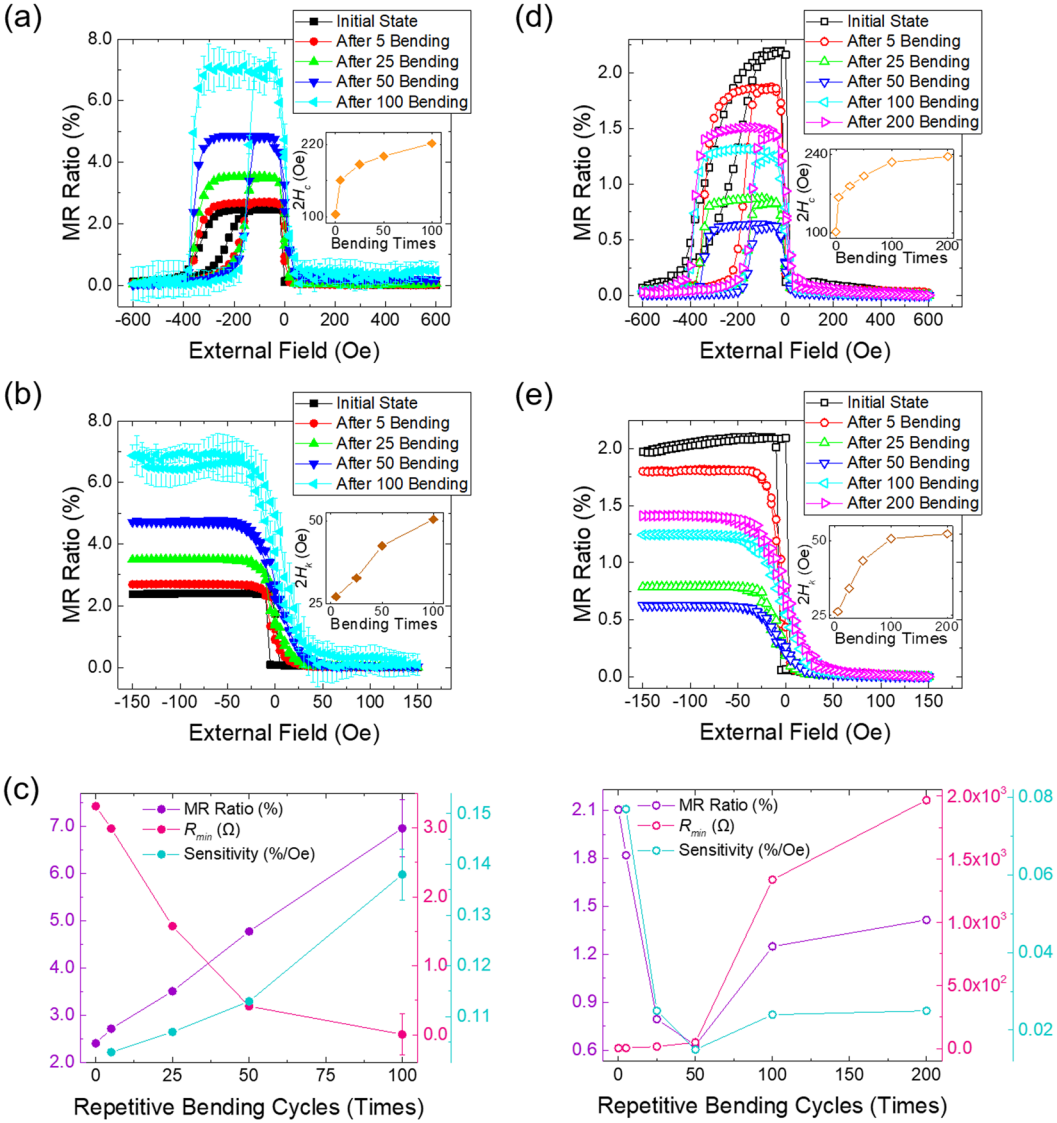
(**a**) and (**b**) Full and minor MR ratio loops in the flat state after 5, 25, 50, and 100-times repetitive bending, respectively, for current flows along the cracks (solid symbols). Insets of (**a**) and (**b**) show the change in 2*H*_*c*_ for the pinned layer (yellow symbols) and 2*H*_*k*_ of the free layer (brown symbols) in terms of the number of bending cycles, respectively. (**c**) Change in the MR ratio (purple symbols), *R*_*min*_ (magenta symbols), and sensitivity (turquoise symbols) as the number of bending cycles increases. (**d**) and (**e**) Full and minor MR ratio loops in the flat state after 5, 25, 50, 100, and 200-times repetitive bending, respectively, for current flow across the cracks (open symbols). Insets of (**d**) and (**e**) also show the change in 2*H*_*c*_ and 2*H*_*k*_ for the pinned (yellow symbols) and free (brown symbols) layers, respectively, depending on the number of repetitive bending cycles. (**f**) Change in the MR ratio (purple symbols), *R*_*min*_ (magenta symbols), and sensitivity (turquoise symbols) with respect to number of repetitive bending cycles.

Figure 4 shows the MR change in terms of the number of bending cycles when the current is applied along (a, b, and c) and transverse (d, e, and f) to the cracks shown in Fig. 2(b). The MR measurement is carried out using a standard 4-point probe system with a current of 10 mA. The external magnetic field is swept along the magnetic easy axis from −600 Oe to 600 Oe with a step size of 20 Oe for full loops and from −150 Oe to 150 Oe with a step of 5 Oe for minor loops. Figure 4(a,b) show the full and minor loops, respectively, for the MR ratio in the after-flat-state with 0, 5, 25, 50, and 100-times bending for the current flowing along the cracks. The MR ratio is expressed as Δ*R/R*_*min*_, where Δ*R* is the resistance difference between two parallel (or anti-parallel) ferromagnetic layers and *R*_*min*_ is the minimum resistance. The shape of the MR loops and its change with the number of bending cycles is consistent with the magnetization curves shown in Fig. 2(c). The inset of Fig. 4(a) shows that 2*H*_*c*_ for the pinned layer increases from 104 Oe in the initial-flat-state to 221 Oe in the after-flat-state for 100-times bending. The inset of Fig. 4(b) shows also that the hysteresis loop of the free layer is gradually tilted, with the 2*H*_*k*_ values increasing from 27 Oe in the initial-flat-state to 50 Oe in the after-flat-state for 100-times bending. Such a change in the hysteresis loop for the transfer curve in a low field range demonstrates magnetic anisotropy relaxation in the free layer. In addition, this change in MR behaviour for the free layer enables one to use the spin-valve as a field sensor as well as the 2*H*_*k*_ values can be increased according to the number of repetitive bending, which means that the operating range of the magnetic sensor can be varied.

Figure 4(c) shows the sensitivity, MR ratio and *R*_*min*_ for the spin-valve sensor, extracted from Fig. 4(a,b), in terms of the number of bending cycles. The MR ratio of 2.3% measured in the initial-flat-state gradually increases to 7.0% in the after-flat state for 100-times bending due to the enhanced magnetic anisotropy of the pinned layer in spite of the reduction of the anisotropy in the free layer. *R*_*min*_ decreases from 3.3 Ω to 0.05 Ω in the after-flat-state for 100-times bending, with the low value of resistance meeting the resolution limit of the equipment used, which leads to a signal error of 11% in the full and minor MR loops, as shown in Fig. 4(a,b). Such a decrease in resistance dependent on repetitive bending seems unrelated to the change in magnetic properties. *R*_*min*_ for similar multilayers without magnetic layers shows the same tendency as that in Fig. 4(c) layers (See Figs S2 and S3 in Supporting Information). The sensitivity, which is defined as the value of MR ratio divided by the switching field interval, which can be expressed as 2*H*_*k*_^40^, also increases monotonically from 0.103%/Oe in the after-flat-state for 5-times bending to 0.138%/Oe in the after-flat-state for 100-times bending because the MR ratio increases rapidly, despite 2*H*_*k*_ also increasing.

Figure 4(d) and e show the full and minor loops, respectively, for the MR ratio in the after-flat-state for 0, 5, 25, 50, 100, and 200-times bending with the current flowing transverse to the cracks. The 2*H*_*c*_ values for the pinned layer increase from 102 Oe to 237 Oe and the 2*H*_*k*_ values for the free layer also increase from 26 Oe to 52 Oe in the after-flat-state for 200-times bending as shown in the inset of Fig. 4(d,e), respectively. Changes in 2*H*_*c*_ and 2*H*_*k*_ depending on the number of bending cycles are quite similar whether the current flows parallel or transverse to the cracks. However, the MR ratio and sensitivity for current flow transverse to the cracks are overall much smaller compared to current flow along the cracks. Figure 4(f) shows the sensitivity, MR ratio, and *R*_*min*_ for the spin-valve sensor, extracted from Fig. 4(d,e) in terms of the number of bending cycles. The MR ratio, which is 2.3% in the initial-flat-state, reaches a local minimum of 0.6% in the after-flat-state for 50-times bending and then increases up to 1.4% in the after-flat-state for 200-times bending. The sensitivity shows a similar bending number dependence with 0.068%/Oe in the after-flat-state for 5-times bending, local minimum of 0.014%/Oe in the after-flat-state for 50-times bending, and 0.027%/Oe in the after-flat-state for 200-times bending. Compared with the results shown in Fig. 4(c), the values for the MR ratio and sensitivity for current flow transverse to the cracks are much smaller than those for current flow along the cracks. On the other hand, *R*_*min*_ values increase slowly from 3.3 Ω in the after-flat-state for 5-times bending to 48.8 Ω in the after-flat-state for 50-times bending and then increase rapidly to 1967.4 Ω in the after-flat-state for 200-times bending. Therefore, we infer that the reduction in the MR ratio and sensitivity for current flow transverse to the cracks is closely related to the nature of the electron transport. The complicated bending-number dependence for the three quantities in Fig. 4(f) is confirmed to be reproducible and cannot be currently understood by a straightforward picture. Detailed information for the mechanical change of the cracks and its relation with electron transport is described in Supporting Information.

### Verification of magnetic beads detection performance of a prototype spin-valve sensor

**Figure 5:**
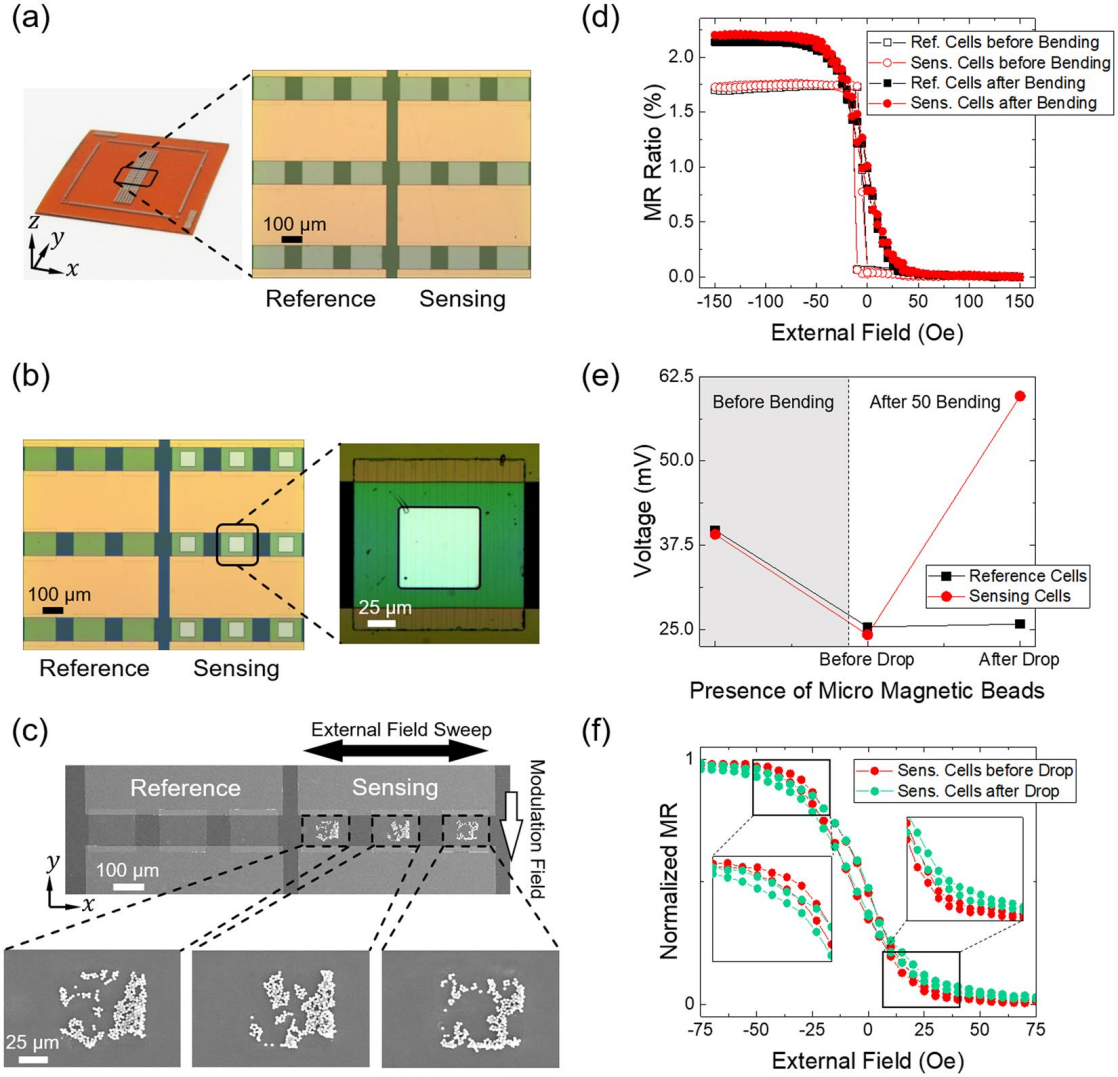
(**a**) Optical images for a spin-valve sensor cell array on a PI substrate. Magnified image on the right side shows a central part of the sensor cell array. The large yellow horizontal rectangles and small green squares are the contact pads and cells, respectively. (**b**) Optical images of the reference cells and sensing cells, with a window for placement of magnetic microbeads onto the centre of the array. The image on the right side shows an enlarged sensing cell with cracks after 50-times bending process. (**c**) SEM images for the 2.8-μm magnetic microbeads on the surface of the sensing cells. Arrows indicate the directions of external sweeping and modulation fields. (**d**) Minor MR ratio loops for the reference cells (black square symbols) and sensing cells (red circle symbols) before bending (open symbols) and after 50-times bending (solid symbols). (**e**) Voltage changes for the reference and sensing cells at zero sweeping field along the *x*-axis. The symbols in the grey and white background areas indicate data in the initial state before bending and in the flat state after 50-times bending, respectively. (**f**) Normalized MR ratio loops for sensing cells before (red circle symbols) and after (green circle symbols) introduction of the magnetic microbeads. Enlarged parts clearly show a shift in the measured loops.

We fabricated a sensor cell array on a PI substrate, as shown in Fig. 5(a), to confirm the detection performance of the spin-valve sensor and to have sufficient resistance not to deviate from the measurement range by the low resistance. The array is separated into reference and sensing areas, each of which contains three cells. Each cell has a lateral dimension of 150 × 150 μm^2^. The detection voltage is the sum of the signals from all three cells. The cells in the array used for reference and sensing are located at the bending centre of the PI substrate because of the effective stress for the formation of an ideal magnetic configuration for the sensor. After 50-times bending along the *x*-axis, random cracks are found along the *y*-axis, similar to those shown in Fig. 2(b). Figure 5(b) shows a square-type window with a dimension of 75 × 75 μm^2^ in a sensing cell. Photo-resist covers the whole surface of the array except the windows in the sensing area. Magnetic microbeads with a diameter of 2.8 μm are placed onto the whole surface of the array and then removed by lift-off of the photoresist to restrict them only to the window area in the sensing cells. Figure 5(c) shows scanning electron microscopy (SEM) images for the three sensing cells, which clearly show the microbeads on the windows. The number of microbeads left on three sensing cells are around 340 beads, which is considered to be enough to affect sensing cells. Non-uniform distribution of the microbeads is likely due to magneto-static interaction between them. The distribution of microbeads on the sensing cells will affect the detection signal, but it, we believe, would not be a critical factor just for the detection of the presence of microbeads.

Figure 5(d) shows MR ratios for the reference and sensing cells in the initial-flat-state and in the after-flat-state for 50-times bending before the introduction of the microbeads. The DC current of 5 mA was injected along the *y*-axis, which is parallel to the cracks. In order to detect magnetic microbeads, we used *x*-*y* double coil system to apply fields along the *x*- and *y*-axis simultaneously. A DC modulation field of 20 Oe was applied along the – *y*-axis to excite magnetic microbeads, and the sensing field was swept along the *x*-axis in a range of −150 Oe ≤ *H* ≤ 150 Oe with a step of 5 Oe. Before bending, reference and sensing cells show a similar MR ratio of 1.7% and 1.72%, respectively. After 50-times bending, the MR ratio increases to 2.14% for the reference cells and 2.19% for the sensing cells, with a sensitivity of ≈0.044%/Oe estimated for both reference and sensing cells. Figure 5(e) shows the voltage differential (Δ*V*) for reference and sensing cells when only modulation field is applied along the – *y*-axis without the sweeping field along the *x*-axis. Before bending, the voltages for both reference and sensing cells are similar and then decrease from ≈39 mV to ≈25 mV after 50-times bending when current flows along the cracks. After magnetic microbeads drop, almost zero Δ*V* is observed for the case of the reference cells, indicating that the magnetic beads on the reference cells are completely removed by the lift-off process. However, for the case of the sensing cells, Δ*V* is 35.4 mV at the zero external field, which is one of typical evidences for the existence of magnetic beads on the cell window area because the stray field from the beads affects magnetization reversal of the free layer^24,25^. Figure 5f shows normalized MR loops for the sensing cells in a field range of −75 Oe ≤ *H* ≤ 75 Oe before and after adding the microbeads. The slope of the transfer curve decreases slightly after the magnetic beads are placed onto the sensing cells, which causes the curve to shift downward in the positive side and upward in the negative field side, and, as a result, *H*_*k*_ increases. This change is also attributed to the stray field produced by the magnetic beads^26,27^. Therefore, detection of Δ*V* and distinct changes in the transfer curve demonstrate that the spin-valve structure on the PI substrate, which is manipulated by repetitive bending, can be used as a magnetic sensor to detect magnetic particles.

## Summary

In summary, a spin-valve structure composed of a free layer (NiFe) and pinned layer (Ni) with positive and negative magnetostriction properties, respectively, is fabricated on a PI substrate. The magnetic configuration of the magnetic sensor between the free and pinned layer is successfully modified by simple repetitive bending without use of conventional thermal and magnetic treatments. The bending-induced changes in magnetic configuration can be understood in terms of an inverse magnetostriction effect driven by residual compressive stress on the free and pinned layers. As the bending number increases, the MR ratio for the spin-valve sensor increases significantly, resulting also in an increased sensitivity when the current flows along the cracks. In addition, 2*H*_*k*_ increases with the bending number for both current directions, meaning that the operating range of the sensor can be controlled. A prototype spin-valve sensor cell array, which is manipulated by 50-times repetitive bending, is fabricated and used to detect magnetic microbeads. The existence of microbeads on the sensing cells is successfully verified using both SEM imaging and electrical (Δ*V*) and magnetic (Δ*H*_*k*_) signals. This indicates that controlled stress by repetitive bending can efficiently manipulate the magnetic configurations for certain devices, e.g., the magnetic spin-valve sensor.

## Methods

### Magnetic properties measurement

A vibrating sample magnetometer (VSM) from Lake Shore Inc. (Model. 7400-s series) and 4-point probe system with gold contact tips are used for the measurement of magnetic properties. The intervals between tips for measuring continuous film are 2 mm. Current injection and voltage detection are carried out using a Keithley 6221 DC current source and Keithley 2182A nanovoltmeter, respectively. A KEPCO bipolar operational power supply is used for a double coil to apply an external magnetic field. Since resistance is temporarily high immediately after the bending process due to the opened cracks and heating effect caused by mechanical deformation, the measurement is carried out after a delay of a few minutes to stabilize resistance.

### Bending machine

A rotational motion is converted to reciprocate motion using a DC motor, which generates a torque of 7.6 kgf∙cm and output of 7.6 W using a KEPCO bipolar operational power supply. The bending radius is modulated by the operation time of the motor on a millisecond scale.

### Microbeads

Dynabeads® M-280 Streptavidin are used for the prototype magnetic sensor cells. In this manuscript, 0.5 μl microbeads, which disperse uniformly with sonication for 30 seconds, are diluted in 5 μl water, which corresponds to a microbead concentration of 0.9 mg/ml. To excite microbeads, a DC modulation field of 20 Oe was applied along the – *y*-axis with external field being swept along the *x*-axis, as shown in Fig. 5(c).

## Electronic supplementary material

Supplementary Information
