# Publisher Correction: Magnetization Manipulation of a Flexible Magnetic Sensor by Controlled Stress Application

**DOI:** 10.1038/s41598-018-36017-8

**Published:** 2018-11-29

**Authors:** Joon-Hyun Kwon, Won-Young Kwak, Beong Ki Cho

**Affiliations:** School of Materials Science and Engineering, Gwangju Institute of Science and Technology, Gwangju, 61005 Republic of Korea

Correction to: *Scientific Reports* 10.1038/s41598-018-34036-z, published online 25 October 2018

The original version of this Article contained a typographical error in the spelling of the corresponding author Beong Ki Cho, which was incorrectly given as Beong Ki. Cho. This has now been corrected in the PDF and HTML versions of the Article.

