# Supplementary material for: Magnetization Manipulation of a Flexible Magnetic Sensor by Controlled Stress Application
Source: Sci Rep. 2018 Oct 25;8:15765. doi: 10.1038/s41598-018-34036-z (PMC6202418; doi:10.1038/s41598-018-34036-z)
Supplement: Supplementary file 1 — Supplementary Information [file 41598_2018_34036_MOESM1_ESM.docx]

Supporting Information

Magnetization Manipulation of a Flexible Magnetic Sensor by Controlled Stress Application

Joon-Hyun Kwon^1^, Won-Young Kwak^1^, and Beong Ki Cho^*,1^

^1^School of Materials Science and Engineering, Gwangju Institute of Science and Technology, Gwangju 61005, Republic of Korea

**1. Comparison of MR loops for spin-valve structures depending on substrates**

Figure S1a and b plot full and minor MR ratio loops, respectively, for spin-valves deposited on a Si/SiO_2_ wafer and PI film. The MR ratio loop for the PI film is the same as the initial loop in Fig. 3. As shown in Fig. S1a, the MR loop for the pinned layer reversal of the spin-valve on PI film is more inclined than that for the spin valve on the Si/SiO_2_ wafer. This can be attributed to the difference in the surface morphology between the Si/SiO_2_ wafer and PI film. The MR ratio, squareness in the minor loop, and 2*H_c_* are all identical for the spin valves on both substrates. The MR ratio is equal to be 2.3%.


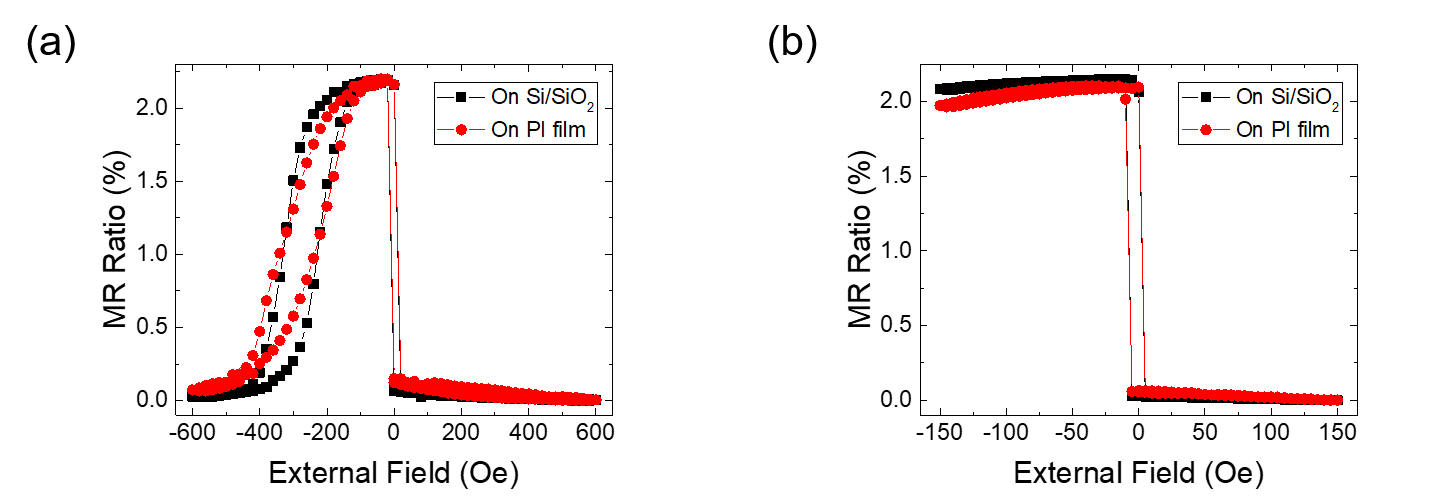


**Figure S1.** (a) and (b) Full and minor MR ratio loops, respectively, for spin-valves on a Si/SiO_2_ wafer (black square symbols) and PI film (red circle symbols).

**2.** **Resistance change depending on the relative orientation of the current flow to the cracks (Experiment)**

Figure S2a shows an optical image for the surface of the stack without magnetic NiFe and Ni layers in the spin-valve structure after 200-times repetitive bending. The width of the cracks lies in a range between 20 and 500 nm, and the distance between the cracks lies in a range between 2 and 8 μm. Moreover, cracks are straight and formed along the direction orthogonal to bending, which are quite similar to those in Fig. 2b. Figure S2b shows the resistance change for the spin-valve structure in terms of the number of bending cycles for current flow parallel or transverse to the cracks without an external magnetic field. The intervals between the 4-point probes are 600 μm, and the initial resistance is measured to be 11 Ω. However, after 200-times bending, the resistance increases to 1960 Ω for current flow across the cracks, whereas the resistance decreases to 2 Ω for current flow along the cracks. Figures S2c and d show the same plot as S2a for 30-nm-thick Cu and Ta layers, respectively. Although the resistance values are found to be different depending on the materials used, changes in resistance in terms of the number of bending cycles show a similar behaviour for all three cases. In addition, the characteristics shown in Fig. S2 are also similar to the change in *R_min_* shown in Fig. 3. Therefore, the observed resistance changes for current flow either along or transverse to the cracks are not due to the magnetic configuration of the ferromagnetic layers or the external magnetic field.


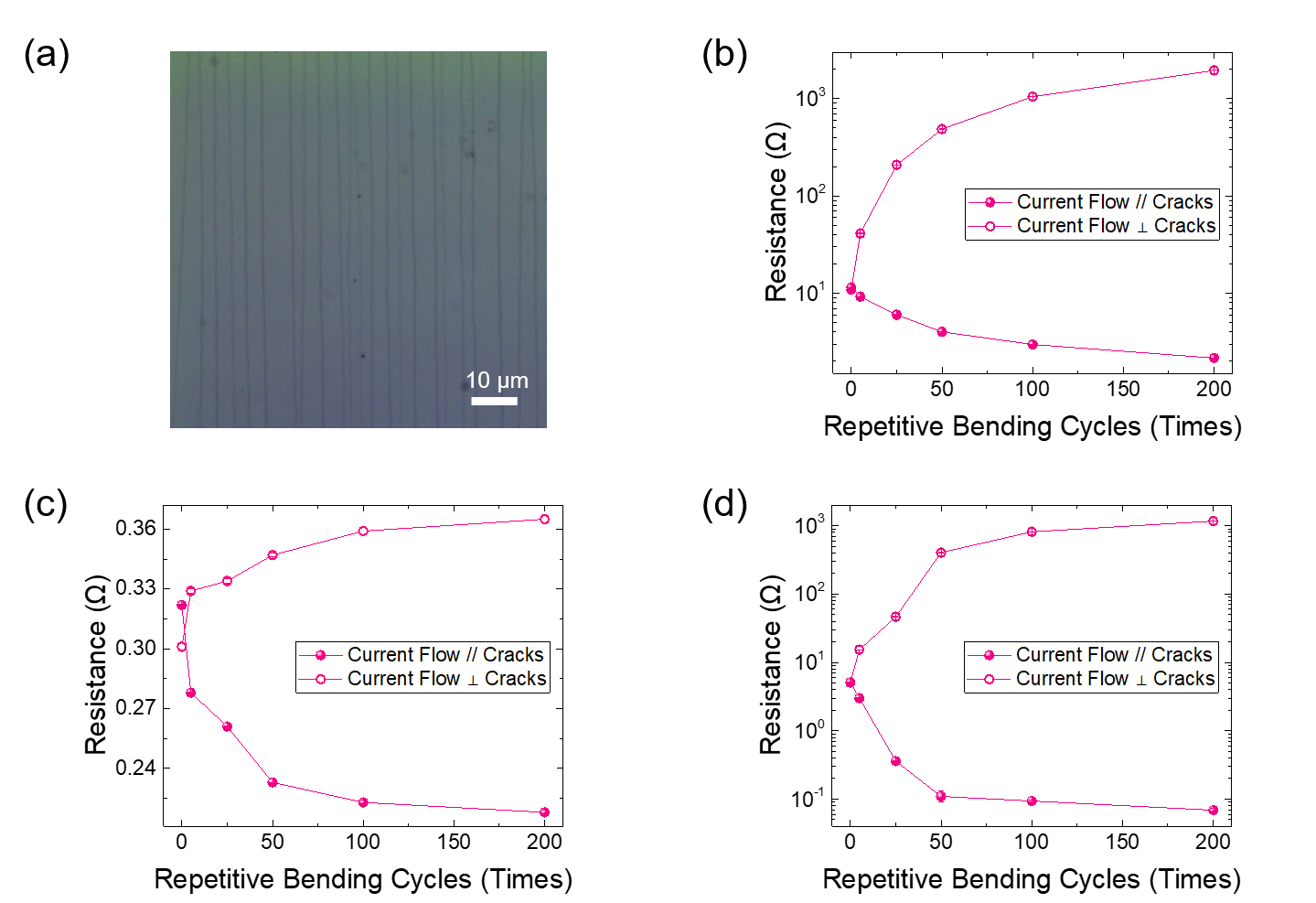


**Figure S2.** (a) Optical image for the top surface of the stack without magnetic NiFe and Ni layers in the spin-valve structure after 200-times repetitive bending. (b) Resistance change for the spin-valve stack in terms of number of bending cycles for current flow parallel or transverse to the cracks. (c) and (d) The same plots as (a) for Cu and Ta layers, respectively.

**3. Current density** **change depending on the relative orientation of current flow to the cracks (Simulation)**

We carried out a simulation for the current density change depending on the relative orientation of the current flow to the cracks using finite element analysis (FEA) with ANSYS AIM and QuickField. The elements in Figs. S3a and d are set to a Cu layer with an area and thickness of 600 × 600 μm^2^ and 30 nm to accommodate the condition in Fig. S2c. Current flows along the *y*-axis in Figs. S3a-c and the *x*-axis in Figs. S3d-f as indicated by a big arrow, respectively. 1, 3 and 5 cracks with a width of 20, 100, and 500 nm are straight along the *y*-axis with a uniform distribution in Figs. S3a and d since straight cracks were formed on the entire surface as shown in Figs. 5b and S2b. All cracks has a gap of 500 nm from the boundaries to allow current to flow through the entire surface. Figures S3a shows the changes in current density when the current flows along the crack, which is calculated by ANSYS AIM, and Fig. S3b shows the current density along the centre red line of the inserted schematics, which are extracted from results of S3a. The current density without cracks is 5.555 × 10^8^ A/m^2^, however, increases to 5.579 × 10^8^ A/m^2^ when there were five cracks with a width of 500 nm. In addition, as shown in Fig. S3a, it is confirmed that the current flow and density are affected at the top and bottom edges of cracks. For a more detailed analysis, we set to a 100-nm-width crack on a 30-nm-thick Cu film with an area of 10 × 10 μm^2^, as shown in the bottom of Fig. S3c, and calculated through QuickField. As a result, the top of Fig. S3c without cracks has the same value of 5.555 × 10^8^ A/m^2^ as the result of Fig. S3b, whereas the overall current density is increased with a crack, especially at the top and bottom edges of the crack.

Figures S3d-f show the change in current density, when the current flows across the cracks. Under the same element conditions as in Figs. S3a-c, Figs. S3d and e are calculated by ANSYS AIM and Fig. S3f is calculated by QuickField. As the number of cracks increases, the current density decreased significantly as shown in Figs. S3d and e. From the simulation results in Fig. S3f, the current density is found to be extremely low in the central area since the current flow is concentrated at the top and bottom edges of the crack, which is consistent with the recent report.^S1^ As a result, the number of cracks and the orientation of the current flow relative to the cracks induces a local and overall current density change, equivalently, resistance changes, which is consistent with experimental results shown in Fig. S2.


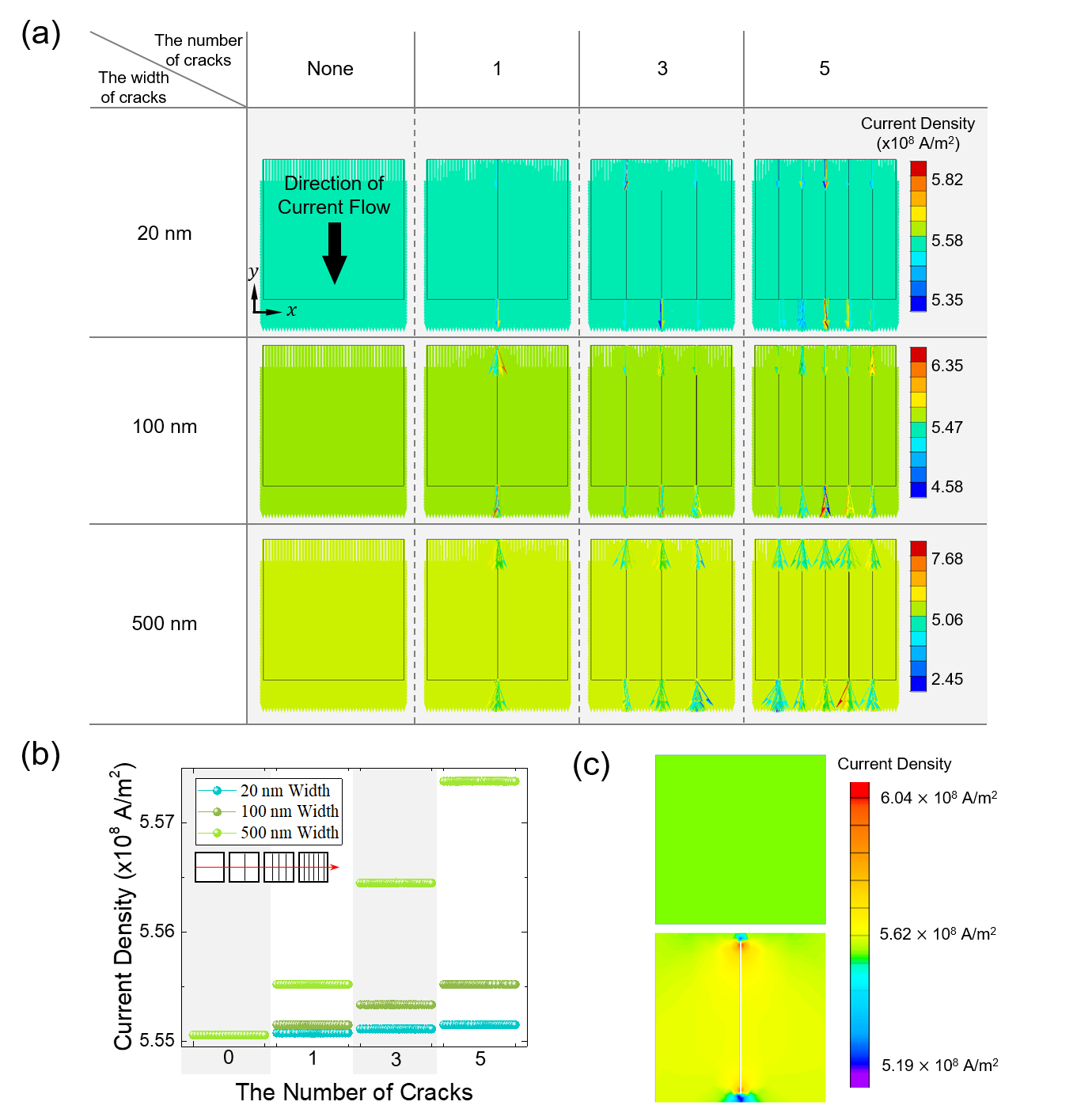


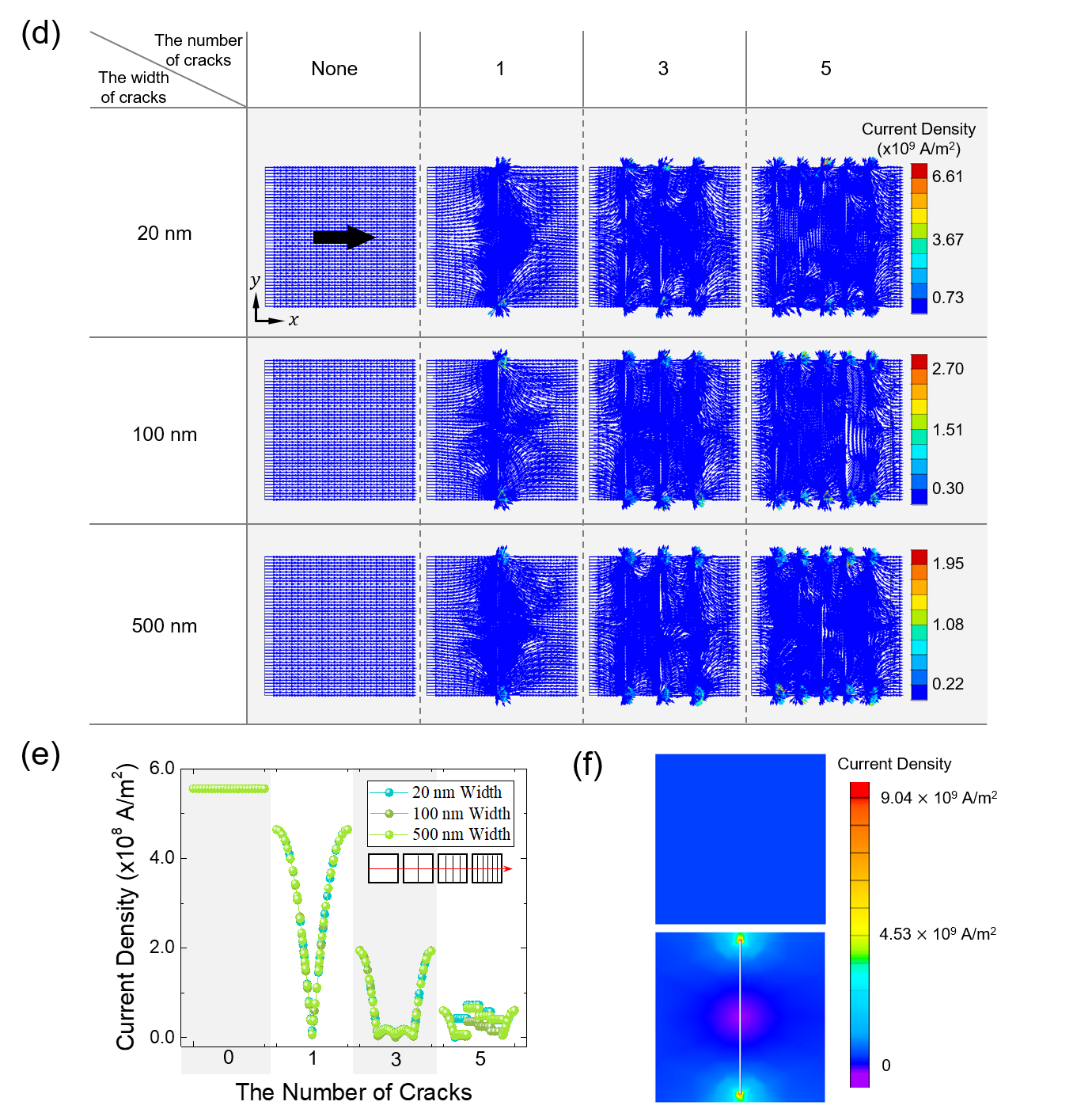


**Figure S3.** Simulation for current density when current flows along the *y*-axis (a, b, c) and the *x*-axis (d, e, f). (a, b) and (d, e) The change of current density calculated by the finite element analysis (FEA) of ANSYS AIM for 30-nm-thick Cu film with an area of 600 × 600 μm^2^. The change in vector (a, d) and value (b, e) of current density depending on the number and width of cracks. The values in (b) and (e) are extracted from results of (a) and (d) along the centre of the surface indicated by a red line of the inserted schematics. (c, f) The contour change of current density simulated by the QuickField FEA for the 30-nm-thick Cu film with an area of 10 × 10 μm^2^ having a crack with a width of 100 nm.

**4. Change in magnetic properties for the spin-valve structures with respect to the number of repetitive bending cycles**

Figures S4a and b show changes in 2*H_c_* for the pinned layer and 2*H_k_* for the free layer, extracted from Fig. 4 with respect to the number of repetitive bending cycles, respectively. Since 2*H_c_* and 2*H_k_* are estimated from the magnetic data, their variation is similar regardless of the orientation of the current flow.


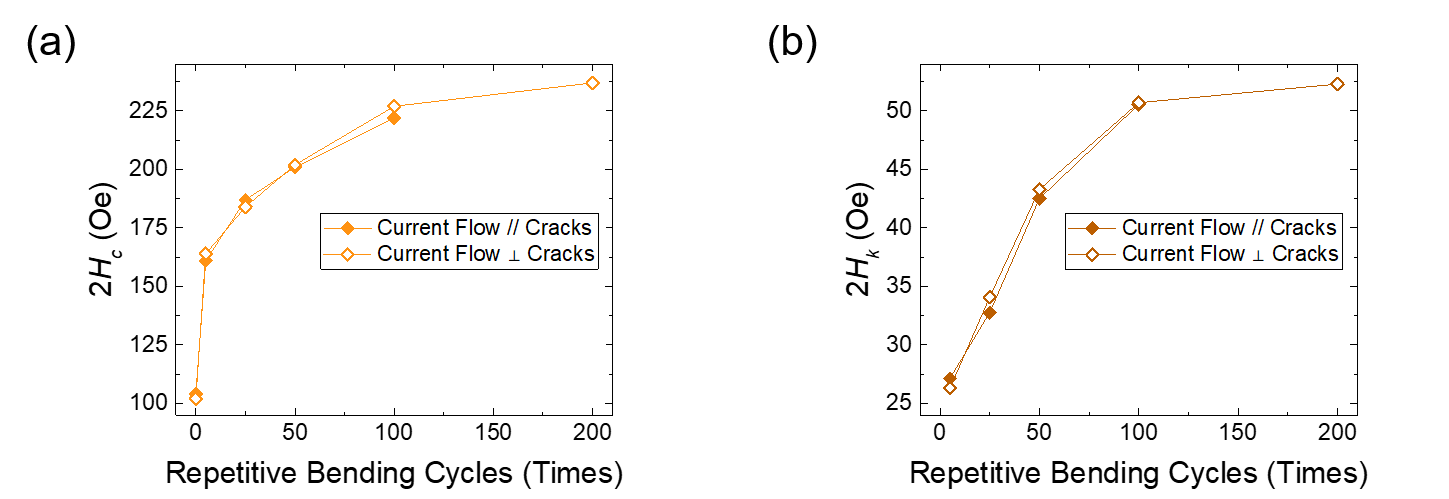


**Figure S4.** (a) and (b) Changes in 2*H_c_* for the pinned layer and 2*H_k_* for the free layer with respect to the number of repetitive bending cycles, respectively, when the current flows along the cracks (solid symbols) and across the cracks (open symbols).

Reference

S1. Graudejus, O. *et al.* The effects of bending on the resistance of elastically stretchable metal conductors, and a comparison with stretching. *Appl. Phys. Lett.* **110,** 221906 (2017).
